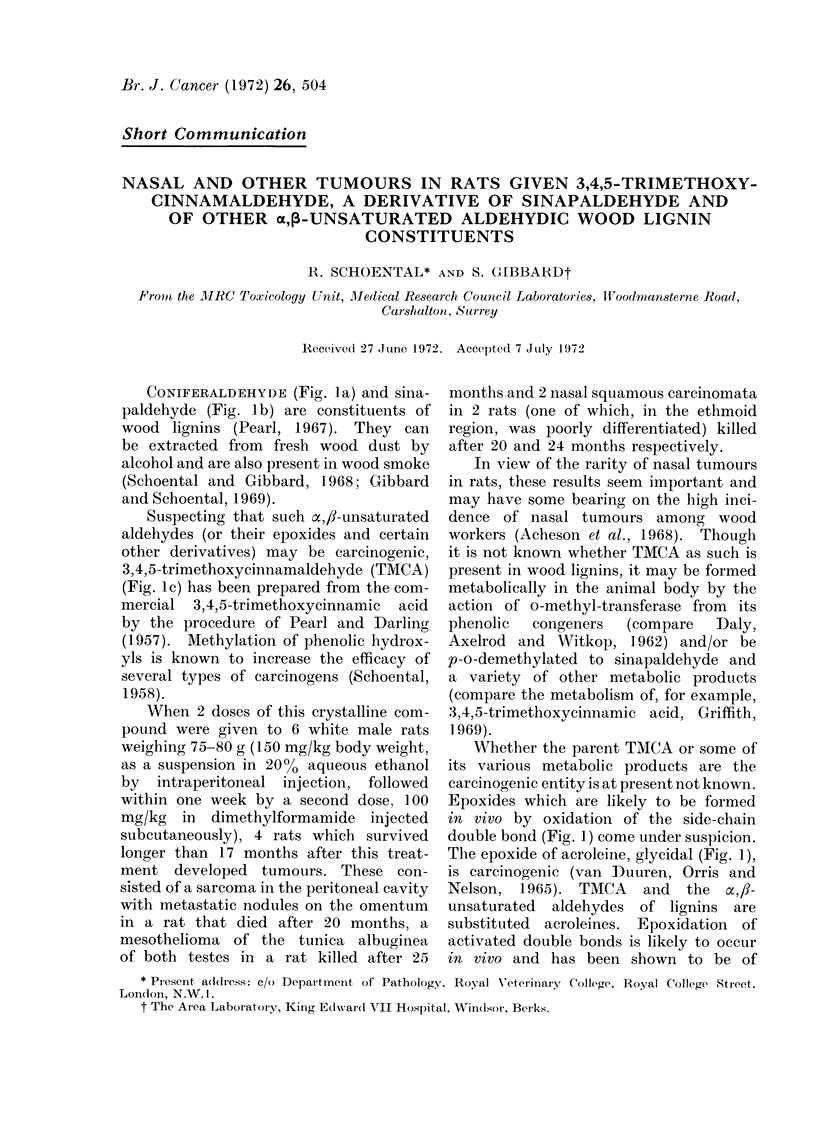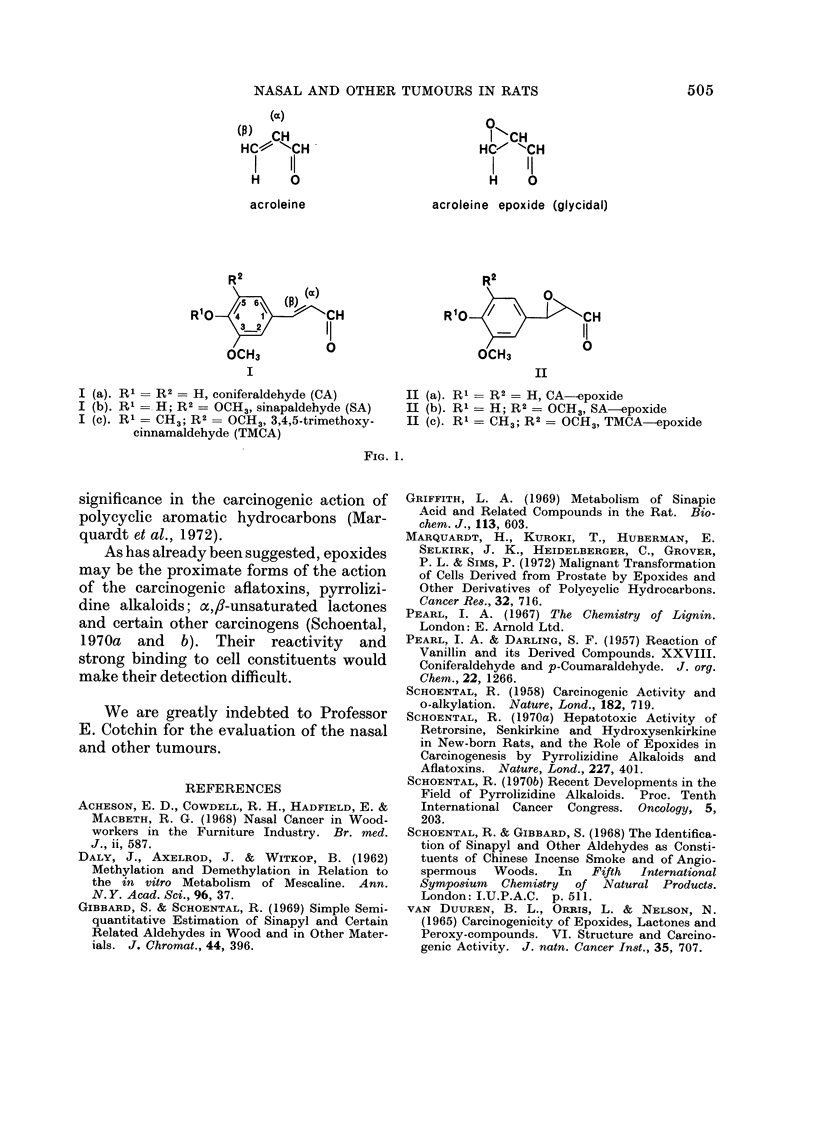# Nasal and other tumours in rats given 3,4,5-trimethoxy-cinnamaldehyde, a derivative of sinapaldehyde and of other , -unsaturated aldehydic wood lignin constituents.

**DOI:** 10.1038/bjc.1972.68

**Published:** 1972-12

**Authors:** R. Schoental, S. Gibbard


*Br. J. Cancer* (1972) **26**, 504

***Short Communication***

### NASAL AND OTHER TUMOURS IN RATS GIVEN 3,4,5-TRIMETHOXY-CINNAMALDEHYDE, A DERIVATIVE OF SINAPALDEHYDE AND OF OTHER α,β-UNSATURATED ALDEHYDIC WOOD LIGNIN CONSTITUENTS

R. SCHOENTAL* AND S. GIBBARD†

*From the MRC Toxicology Unit, Medical Research Council Laboratories, Woodmansterne Road, Carshalton, Surrey*

Received 27 June 1972. Accepted 7 July 1972

CONIFERALDEHYDE (Fig. 1a) and sinapaldehyde (Fig. 1b) are constituents of wood lignins (Pearl, 1967). They can be extracted from fresh wood dust by alcohol and are also present in wood smoke (Schoental and Gibbard, 1968; Gibbard and Schoental, 1969).

Suspecting that such $\alpha,\beta$-unsaturated aldehydes (or their epoxides and certain other derivatives) may be carcinogenic, 3,4,5-trimethoxycinnamaldehyde (TMCA) (Fig. 1c) has been prepared from the commercial 3,4,5-trimethoxycinnamic acid by the procedure of Pearl and Darling (1957). Methylation of phenolic hydroxyls is known to increase the efficacy of several types of carcinogens (Schoental, 1958).

When 2 doses of this crystalline compound were given to 6 white male rats weighing 75–80 g (150 mg/kg body weight, as a suspension in 20% aqueous ethanol by intraperitoneal injection, followed within one week by a second dose, 100 mg/kg in dimethylformamide injected subcutaneously), 4 rats which survived longer than 17 months after this treatment developed tumours. These consisted of a sarcoma in the peritoneal cavity with metastatic nodules on the omentum in a rat that died after 20 months, a mesothelioma of the tunica albuginea of both testes in a rat killed after 25 months and 2 nasal squamous carcinomata in 2 rats (one of which, in the ethmoid region, was poorly differentiated) killed after 20 and 24 months respectively.

In view of the rarity of nasal tumours in rats, these results seem important and may have some bearing on the high incidence of nasal tumours among wood workers (Acheson *et al.*, 1968). Though it is not known whether TMCA as such is present in wood lignins, it may be formed metabolically in the animal body by the action of o-methyl-transferase from its phenolic congeners (compare Daly, Axelrod and Witkop, 1962) and/or be *p*-o-demethylated to sinapaldehyde and a variety of other metabolic products (compare the metabolism of, for example, 3,4,5-trimethoxycinnamic acid, Griffith, 1969).

Whether the parent TMCA or some of its various metabolic products are the carcinogenic entity is at present not known. Epoxides which are likely to be formed *in vivo* by oxidation of the side-chain double bond (Fig. 1) come under suspicion. The epoxide of acroleine, glycidal (Fig. 1), is carcinogenic (van Duuren, Orris and Nelson, 1965). TMCA and the $\alpha,\beta$-unsaturated aldehydes of lignins are substituted acroleines. Epoxidation of activated double bonds is likely to occur *in vivo* and has been shown to be of

* Present address: c/o Department of Pathology, Royal Veterinary College, Royal College Street, London, N.W.1.

† The Area Laboratory, King Edward VII Hospital, Windsor, Berks.

acroleine acroleine epoxide (glycidal)

I

II

I (a). $R^1 = R^2 = H$, coniferaldehyde (CA)
I (b). $R^1 = H$; $R^2 = OCH_3$, sinapaldehyde (SA)
I (c). $R^1 = CH_3$; $R^2 = OCH_3$, 3,4,5-trimethoxy-cinnamaldehyde (TMCA)

II (a). $R^1 = R^2 = H$, CA—epoxide

II (b). $R^1 = H$; $R^2 = OCH_3$, SA—epoxide

II (c). $R^1 = CH_3$; $R^2 = OCH_3$, TMCA—epoxide

FIG. 1.

significance in the carcinogenic action of polycyclic aromatic hydrocarbons (Marquardt *et al.*, 1972).

As has already been suggested, epoxides may be the proximate forms of the action of the carcinogenic aflatoxins, pyrrolizidine alkaloids; $\alpha,\beta$-unsaturated lactones and certain other carcinogens (Schoental, 1970*a* and *b*). Their reactivity and strong binding to cell constituents would make their detection difficult.

We are greatly indebted to Professor E. Cotchin for the evaluation of the nasal and other tumours.

### REFERENCES

ACHESON, E. D., COWDELL, R. H., HADFIELD, E. & MACBETH, R. G. (1968) Nasal Cancer in Woodworkers in the Furniture Industry. *Br. med. J.*, ii, 587.

DALY, J., AXELROD, J. & WITKOP, B. (1962) Methylation and Demethylation in Relation to the *in vitro* Metabolism of Mescaline. *Ann. N.Y. Acad. Sci.*, **96**, 37.

GIBBARD, S. & SCHOENTAL, R. (1969) Simple Semiquantitative Estimation of Sinapyl and Certain Related Aldehydes in Wood and in Other Materials. *J. Chromat.*, **44**, 396.

GRIFFITH, L. A. (1969) Metabolism of Sinapic Acid and Related Compounds in the Rat. *Biochem. J.*, **113**, 603.

MARQUARDT, H., KUROKI, T., HUBERMAN, E. SELKIRK, J. K., HEIDELBERGER, C., GROVER, P. L. & SIMS, P. (1972) Malignant Transformation of Cells Derived from Prostate by Epoxides and Other Derivatives of Polycyclic Hydrocarbons. *Cancer Res.*, **32**, 716.

PEARL, I. A. (1967) *The Chemistry of Lignin.* London: E. Arnold Ltd.

PEARL, I. A. & DARLING, S. F. (1957) Reaction of Vanillin and its Derived Compounds. XXVIII. Coniferaldehyde and *p*-Coumaraldehyde. *J. org. Chem.*, **22**, 1266.

SCHOENTAL, R. (1958) Carcinogenic Activity and o-alkylation. *Nature, Lond.*, **182**, 719.

SCHOENTAL, R. (1970*a*) Hepatotoxic Activity of Retrorsine, Senkirkine and Hydroxysenkirkine in New-born Rats, and the Role of Epoxides in Carcinogenesis by Pyrrolizidine Alkaloids and Aflatoxins. *Nature, Lond.*, **227**, 401.

SCHOENTAL, R. (1970*b*) Recent Developments in the Field of Pyrrolizidine Alkaloids. Proc. Tenth International Cancer Congress. *Oncology,* **5**, 203.

SCHOENTAL, R. & GIBBARD, S. (1968) The Identification of Sinapyl and Other Aldehydes as Constituents of Chinese Incense Smoke and of Angiospermous Woods. In *Fifth International Symposium Chemistry of Natural Products.* London: I.U.P.A.C. p. 511.

VAN DUUREN, B. L., ORRIS, L. & NELSON, N. (1965) Carcinogenicity of Epoxides, Lactones and Peroxy-compounds. VI. Structure and Carcinogenic Activity. *J. natn. Cancer Inst.*, **35**, 707.